# Building recurring public and patient involvement in adult autism psychiatry and neurodevelopmental research: lessons from the first year of a patient panel and advisory board

**DOI:** 10.1186/s40900-026-00990-6

**Published:** 2026-10-01

**Authors:** Kerstin Erdal, Tomas Larson, Eva Billstedt

**Affiliations:** 1 Department of Neuropsychiatry, Sahlgrenska University Hospital, Gothenburg, Region Västra Götaland Sweden; 2 Department of Psychiatry and Neurochemistry, Institute of Neuroscience and Physiology, University of Gothenburg, Gothenburg, Sweden; 3 Gillberg Neuropsychiatric Centre, Institute of Neuroscience and Physiology, University of Gothenburg, Gothenburg, Sweden; 4 Child and Adolescent Neuropsychiatric Clinic, Queen Silvia Children’s Hospital, Gothenburg, Sweden; 5 Sahlgrenska Academy, Medicinaregatan 3, Gothenburg, 413 90 Sweden

**Keywords:** Public and patient involvement, Participatory research, Autism, Psychiatry, Neurodevelopmental research

## Abstract

Public and patient involvement (PPI) is increasingly emphasized in health and autism research, yet involvement is often organized around individual research projects. Less is known about the practical and organizational processes involved in developing recurring PPI structures across clinical and academic settings. This paper describes and critically reflects on the establishment, implementation, and first-year experiences of two recurring PPI structures in western Sweden: a Patient Panel linked to an adult autism psychiatry service and a Neurodevelopmental Disorders (NDD) Advisory Board based at the University of Gothenburg. The two structures followed different implementation pathways. The Patient Panel was initially anchored to an ongoing research programme and involved autistic adults receiving psychiatric care, whereas the NDD Advisory Board was established from the outset as a broader forum bringing together researchers and representatives of patient and user organizations. First-year activities included review of research materials, ethical and methodological reflection, intervention development, discussion of research findings and clinical service development, and input into broader neurodevelopmental research questions. Contributor input resulted in identifiable changes to research procedures and intervention content, although involvement remained predominantly advisory and final decision-making authority remained with researchers, project leads, or clinical management. Implementation highlighted the importance of institutional context, organizational support, preparation, accessibility, compensation, and mechanisms for follow-up. Establishing the clinically embedded Patient Panel required iterative development of procedures concerning recruitment, data protection, compensation, and organizational responsibilities, whereas the university-based Advisory Board was administratively more straightforward to establish. Across both structures, governance, systematic documentation and follow-up of recommendations, and shared decision-making remained underdeveloped. Considered in relation to AASPIRE principles, several participatory practices were established, while important gaps remained in governance, accessibility procedures, and involvement across research stages. These experiences demonstrate that recurring PPI activities can be established in both clinical and academic settings, while highlighting that their establishment should not be equated with long-term sustainability or institutionalization. Developing sustained PPI requires ongoing attention to organizational procedures, resources, accountability, accessibility, and the distribution of decision-making authority.

**Supplementary Information:**

The online version contains supplementary material available at https://doi.org/10.1186/s40900-026-00990-6.

## Clinical trial number

Not applicable.

**Supplementary Information:**

The online version contains supplementary material available at https://doi.org/10.1186/s40900-026-00990-6.

## Abstract (plain language summary)

Patients and members of the public are increasingly invited to contribute to health research. However, this involvement is often connected to only one research project. We wanted to explore ways of creating groups that could meet repeatedly and contribute to a range of research and healthcare questions over time. We describe the first year of two such groups in western Sweden. The first is a Patient Panel connected to a psychiatric service for autistic adults. The second is an advisory group at the University of Gothenburg that brings together researchers and representatives of patient and user organizations working with questions related to neurodevelopmental conditions. The two groups developed differently. The Patient Panel started in connection with one research programme and later became involved in other research and healthcare-development questions. The university advisory group included researchers from different departments. Contributors from both the Patient Panel and the NDD Advisory Board provided input, although their roles differed. Patient Panel members contributed to research materials, development of a clinical group intervention, discussion of research findings, and healthcare service-development questions. The NDD Advisory Board contributed particularly to ethical and methodological questions and to broader neurodevelopmental research topics. Some suggestions resulted in concrete changes. However, both groups mainly had an advisory role: researchers and clinical leaders continued to make the final decisions. The first year also showed that creating recurring involvement requires practical work. Recruitment, compensation, preparation, accessibility, clear responsibilities, and communication about what happens after members provide advice all need to be considered. These experiences show that recurring involvement can be established in both clinical and university settings, but one year is too early to know whether these groups will be sustainable over the longer term.

**Supplementary Information:**

The online version contains supplementary material available at https://doi.org/10.1186/s40900-026-00990-6.

## Introduction

Public and patient involvement (PPI) and participatory research approaches have become increasingly central within health and disability research, emphasizing lived experience as a legitimate and necessary form of expertise. In this paper, PPI refers to the active involvement of patients and community stakeholders in shaping research and service development, rather than participation as research subjects. PPI may encompass varying levels of involvement, ranging from consultation and advisory input to collaborative co-design and co-production, in which decision-making authority is shared between researchers and community stakeholders [2, 12].

In this paper, we describe the establishment and early implementation of two recurring PPI structures intended to support sustained collaboration between researchers, clinicians, patients, and community stakeholders across research and service-development activities. Throughout this paper, the term *patient* is used specifically in relation to individuals receiving care from the participating psychiatric service. When referring to their role as contributors within the Patient Panel, we use the term *Panel members*. Researchers, clinicians, organizational representatives, and moderators participating in Panel activities are described according to their respective roles. The term *Neurodevelopmental Disorders (NDD)* reflects the institutional and clinical terminology used when the NDD Advisory Board was established.

In autism research, calls for participatory research have intensified in response to longstanding epistemic asymmetries [3, 8, 13]. Participatory and co-designed methods are now widely regarded best practice in autism research and are recommended to enhance epistemic justice, following the programmatic claim of ‘no research on us without us’ [1, 12]. Beyond epistemic considerations, empirical evidence suggests that participatory approaches can enhance research relevance, improve interpretation, and strengthen translation into practice [5, 9, 15]. Higher levels of involvement allow stakeholders to shape priorities, influence methodological decisions, and collaboratively interpret findings, addressing both epistemic and ethical concerns related to representation and justice [4, 7]. However, establishing opportunities for involvement does not in itself ensure shared decision-making or equitable influence. The extent to which lived-experience expertise can shape research depends on how roles, authority, accessibility, representation, and institutional responsibilities are negotiated in practice.

A prominent example of participatory autism research is the Academic Autism Spectrum Partnership in Research and Education (AASPIRE), a community-based participatory research collaboration involving autistic adults, researchers, and community stakeholders as co-researchers and co-decision-makers [9, 11]. Building on these experiences, AASPIRE developed practice-based guidelines for the inclusion of autistic adults in research. These guidelines emphasize transparent governance, accessible communication, accommodations, fair compensation, role clarification, and involvement across multiple stages of the research process [10]. The guidelines describe principles for meaningful participatory practice rather than a single model or level of involvement, and their implementation may vary according to the purpose, setting, and stage of development of a participatory initiative. We therefore use the AASPIRE guidelines as a reference framework for reflecting on the development of the two PPI structures, including both areas of alignment and areas where participatory practices remained underdeveloped during the first year.

Against this backdrop, we report on the process of establishing two recurring PPI structures in western Sweden: (1) a Patient Panel linked to an adult autism psychiatry clinic and (2) a Neurodevelopmental Disorders (NDD) Advisory Board at the University of Gothenburg. The aim of this paper is to describe and critically reflect on the establishment, implementation, and first-year experiences of these two PPI structures. Specifically, we describe the process of establishing and operating the structures, the forms of involvement that occurred during the first year, and the practical, relational, and organizational challenges encountered during their early implementation.

## Methods and implementation

This paper presents a descriptive implementation report with a researcher-led reflective component, focusing on the establishment and first year of operation of the Patient Panel and the NDD Advisory Board. The implementation period covered the period from September 2025 – August 2026. The account draws on meeting agendas, minutes, researcher notes, and records of activities and changes generated during operation of the two structures. Internal implementation feedback collected following the second NDD Advisory Board meeting was used as contextual information informing the researcher-led reflection but was not analysed as research data. Reporting of the PPI activities was guided by the GRIPP2-LF checklist [15], which was used as a reporting framework rather than as a method for evaluating the quality or impact of PPI. The implementation records were collated by KE, who had been directly involved in establishing and operating both structures. The records and emerging reflections were reviewed and discussed among the authors during preparation of the manuscript.

### Clinical and institutional context

The participatory initiative developed through collaboration between the adult outpatient autism psychiatry service at Sahlgrenska University Hospital and researchers at the University of Gothenburg. Two complementary structures were established: a Patient Panel within the clinical service and a NDD Advisory Board within the university setting.

The Patient Panel was linked to a publicly funded specialist outpatient service at Sahlgrenska University Hospital providing psychiatric care for autistic adults with co-occurring psychiatric conditions. The service covers Gothenburg and the wider Västra Götaland region in western Sweden and had approximately 200 patients receiving care at the time the Panel was established. The Patient Panel was developed in collaboration with the Gothenburg branch of the Swedish Partnership for Mental Health (NSPH Gothenburg), a regional umbrella organization for patient, service-user, and family organizations within mental health. NSPH provided organizational support, moderated Panel meetings, and acted as an intermediary in recruitment by contacting interested patients after they had agreed that their contact information could be shared with the organization.

The NDD Advisory Board was established within the Institute of Neuroscience and Physiology at the Sahlgrenska Academy, University of Gothenburg. The Board brought together academic researchers and representatives from patient and user organizations, including Autism Sverige, Riksförbund Attention, and the Swedish National Association for People with Intellectual Disability (FUB).

### Researcher positionality

The authors were directly involved in establishing and developing the PPI structures described in this report. All organizers of the NDD Advisory Board are licensed psychologists and researchers. KE, the principal investigator of the research programme initially discussed within the Patient Panel, is affiliated with both the adult autism psychiatry service and the University of Gothenburg. She initiated the Patient Panel, contributed to the establishment of the NDD Advisory Board, and has previous experience with participatory research approaches. EB contributed to the establishment and moderation of the NDD Advisory Board, and TL is clinically affiliated with the adult psychiatry service. The authors therefore occupied overlapping research, clinical, organizational, and reporting roles during implementation. These positions provided direct knowledge of the implementation process but may also have influenced how the structures were developed, which aspects of implementation were prioritized, and how the experiences reported here were interpreted.

### Recruitment and eligibility criteria

#### Patient panel

Recruitment to the Patient Panel was limited to autistic adults receiving care at the adult autism psychiatry clinic. Admission to the clinic requires a clinically confirmed autism spectrum disorder (ASD) diagnosis and a co-occurring psychiatric condition; individuals with intellectual disability are not admitted to the service. Consequently, the Patient Panel did not include autistic adults with intellectual disability. Information about the Panel was made available to patients at the clinic and described its purpose, the voluntary nature of involvement, compensation, and the opportunity to participate without previous research experience or participation as a research subject. Interested patients registered their interest with their clinician or a clinic secretary and provided contact information that they agreed could be shared with NSPH Gothenburg. NSPH subsequently contacted interested individuals regarding participation in the Panel. Recruitment was intentionally restricted to patients of the adult autism psychiatry clinic. Broader recruitment through NSPH Gothenburg was considered but not pursued because the Panel was intended to provide lived-experience perspectives specifically connected to the clinic and its research and service-development activities. Demographic characteristics, communication needs, and previous experience of PPI or research were not systematically collected from Panel members, as these characteristics were not used as eligibility criteria.

#### NDD advisory board

Before establishing the NDD Advisory Board, the initiative was discussed with the Head and Vice-Head of the Institute of Neuroscience and Physiology at the University of Gothenburg, who supported the establishment of the Board within the Institute. The Institute includes several research groups conducting multidisciplinary autism and neurodevelopmental research spanning neurobiology, biomarkers, genetics, social behaviour, and treatment across the lifespan. Recruitment of researchers developed during the first year. For the first meeting, researchers working in neurodevelopmental research were approached directly through relevant research environments and existing academic contacts. For the second meeting, recruitment was broadened within the Institute through an open invitation distributed in the Institute’s newsletter. Researchers interested in bringing a topic to the NDD Advisory Board were asked to contact the organizers in advance and provide a brief description of their research or the topic they wished to discuss, together with specific questions on which they sought input from the Board. Individual demographic or lived-experience characteristics of researchers and organization representatives were not systematically collected; contributors are therefore described according to the roles in which they participated in the Advisory Board.

### Meeting procedures, accessibility, and compensation

#### Patient panel

Three Patient Panel meetings were held during the reporting period. Meetings lasted approximately two to two and a half hours and were held at the NSPH Västra Götaland office in Gothenburg, with the option to participate digitally. Meetings were moderated by a representative from NSPH. Agendas were developed by the research and clinical team around ongoing research and service-development activities rather than co-determined with Panel members. Agenda/topic descriptions circulated approximately two weeks beforehand by email. Meetings included scheduled breaks and refreshments. At the initial meeting, Panel members received an introduction to the clinic’s research programme, the purpose and role of the Patient Panel, and participatory research. Efforts were made to accommodate members’ needs during meetings, for instance through scheduled breaks, sensory accommodations, and, at the second meeting, refreshments accommodating different food preferences and dietary requirements. Panel members were offered different ways of contributing depending on the activity, including oral and written feedback during meetings and the possibility of providing feedback by email after the meeting.

Panel members received compensation for meeting participation in accordance with regional guidelines. Preparation time was not compensated. Feedback on previous activities was provided at subsequent meetings when relevant (see Table 2 for details). However, no formal procedure was in place during the first year for systematically tracking all recommendations or documenting whether and how each recommendation was subsequently implemented.

#### NDD advisory board

Meetings of the NDD Advisory Board were conducted online and lasted approximately 60 min. Meetings were moderated by the organizing researchers. Researchers wishing to bring a project or question to the Board were asked in advance to provide a brief description of the topic and to formulate specific questions on which they sought input. Based on these submissions, the organizers prepared the meeting agenda. For the second meeting, the agenda, including brief descriptions of the presenting researchers, their projects or topics, and the questions to be discussed, was circulated to Board members approximately two weeks before the meeting.

Meetings were structured around brief research presentations followed by discussion with Board members. Members were also encouraged to provide additional feedback by email. Attendance and composition at each meeting are reported in Table 1. No formal procedure for assessing individual accessibility or communication needs was used, and no formal onboarding programme was provided, although new members were introduced at the beginning of meetings. Participation in the NDD Advisory Board was not compensated. Presenting researchers were responsible for taking notes on the feedback relevant to their own projects or questions; no centralized system for recording or tracking recommendations was used. Researchers were encouraged to return to the Board at a later meeting to report how the discussion or recommendations had been used, but no such follow-up had occurred during the reporting period. Table 1 summarizes the different formats and characteristics.

**Table 1:** Characteristics and operation of the patient panel and NDD advisory board

Indicator	Patient Panel	NDD Advisory Board
Institutional setting	Adult autism psychiatry service, Sahlgrenska University Hospital	Institute of Neuroscience and Physiology, University of Gothenburg
Primary contributors	Autistic adults receiving psychiatric care at the adult autism psychiatric service unit	Researchers and representatives of patient/user organisations
Composition	6–9 autistic Panel members with psychiatric care experience; 1 representative from Attention; 1–3 researchers/clinical staff from the adult autism psychiatry service; 1 NSPH representative as moderator	Open, recurring forum without fixed membership; 4–8 researchers involved during the reporting period; one representative each from Attention Sverige, Autism Sverige, and FUB, participating in their organizational representative capacity; organizing researchers served as moderators.
Attendance	Session 1: 5 patient representatives, 1 representative from Attention, 2 researchers, 1 unit manager, 1 moderator from NSPH Session 2: 8 patient representatives, 1 representative from Attention, 3 researchers, 2 moderators from NSPH Session 3: 9 patient representatives, 1 representative from Attention, 2 researchers, 1 unit manager, 1 moderator from NSPH	Session 1: 4 researchers, 2 organizers, 1 representative from Attention, 1 representative from Autism Sverige Session 2: 8 researchers, 2 organizers, 1 representative from Attention, 1 representative from Autism Sverige, 1 representative from FUB.
Organisations represented	NSPH as collaborating/moderating organization	Autism Sverige, Attention, FUB
Recruitment	Open invitation to patients at the clinic; interested patients provided contact information for transfer to NSPH, which subsequently contacted them	Meeting 1: direct approach through relevant research environments/academic contacts; Meeting 2: open invitation to researchers through Institute newsletter; organisation representatives through existing contacts
Eligibility/scope	Autistic adults with experience of psychiatric care at the clinic; research participation/experience not required	Researchers working with relevant NDD questions/projects; representatives of relevant patient/user organisations
Primary focus	Lived-experience input into clinic-related research and service development	Methodological, ethical and broader NDD research questions
Meetings during reporting period	3	2
Meeting duration	Approx. 2–2.5 h	Approx. 60 min
Format	Hybrid	Online
Facilitation	NSPH representative	Organising researchers
Agenda setting	Research/clinical team selected topics	Organisers based on projects/questions submitted by researchers
Advance materials	Agenda/topic descriptions circulated approximately two weeks beforehand	Agenda/topic descriptions circulated approximately two weeks beforehand
Ways of contributing	Oral and written feedback during meetings; email feedback where appropriate	Discussion during meetings; additional email feedback encouraged
Accessibility	Hybrid participation, scheduled breaks, multiple modes of providing feedback; needs accommodated where possible, but no systematic assessment	Online participation; no formal systematic assessment of accessibility/communication needs
Compensation	Meeting participation compensated according to regional guidelines; travel reimbursed when claimed; preparation time not compensated	No compensation
Onboarding/orientation	Initial information about research programme, Panel role and participatory research	Introduction of new members; no formal onboarding
Documentation	Researchers taking notes and receiving written feedback	Presenting researchers took notes relevant to their own project; no central recommendation log
Follow-up	Feedback provided at subsequent meetings where relevant; no systematic tracking of all recommendations	Researchers encouraged to return and report how advice was used; this had not occurred during reporting period
Decision-making authority	Researchers/clinical leadership	Researchers/project leads

Note. NSPH = Swedish Partnership for Mental Health; Attention Sverige, Autism Sverige, and FUB are Swedish patient, service-user, and advocacy organizations representing people with neurodevelopmental conditions and/or intellectual disabilities and their families. Representatives participated in the NDD Advisory Board in their organizational representative capacity

## First-year implementation and PPI activities

The first year represented an early implementation period during which organizational procedures for recurring PPI were developed alongside the activities of the two structures. Neither setting had an established recurring PPI structure of this kind on which to build, and implementation therefore involved developing and adapting procedures while the groups were becoming operational. The nature of this process differed substantially between the clinical and university settings.

Establishing the Patient Panel within the clinical service required repeated organizational and administrative adjustments. The intended scope of the Patient Panel required negotiation during establishment. Our aim was to develop a recurring PPI structure that could contribute to research and service development beyond individual projects. In practice, the Panel was initially established in connection with a concrete ongoing research project examining autistic adults’ experiences of accessing and receiving psychotherapy. Linking the Panel to this project provided an organizational framework through which recruitment, meeting organization, collaboration with NSPH, and compensation procedures could be established. The intention, however, was not to restrict the Panel’s activities to this project, and its remit subsequently broadened as the structure became operational.

One early issue concerned data protection and how interested patients could be contacted without the research team collecting or transferring patient contact information outside established procedures. This was addressed through collaboration with NSPH: interested patients agreed that their email addresses could be shared with NSPH, which subsequently contacted them regarding participation in the Panel.

Administrative procedures for compensation represented another implementation challenge. Regional guidelines for compensating patient representatives were available, but establishing a functioning procedure for applying these guidelines within the Panel required repeated clarification and coordination. Once established, Panel members were compensated according to the regional framework, with travel expenses reimbursed when claimed.

At the same time, anchoring the Panel within the clinical service emerged as an important enabling factor. Clinical management supported the initiative, and interaction between the Panel and the service developed over the reporting period. The Panel’s activities also broadened from input into the qualitative interview study to other research and service-development activities, including the development of a clinical group intervention. At the third meeting, the unit manager provided Panel members with an update on developments within the clinical service, creating an opportunity for direct dialogue about the development of the service.

In contrast, establishment of the NDD Advisory Board within the university setting required fewer administrative adaptations. Following support for the initiative at the Institute level, researchers and representatives of patient and user organizations could be brought together through existing academic and organizational networks. Recruitment of researchers subsequently broadened from direct contacts to an open invitation through the Institute newsletter. The online meeting format, advance submission of research questions, and circulation of agendas provided a relatively straightforward structure for bringing different research projects to the Board.

### Contributions to research and service development

During the reporting period, the two PPI structures contributed to research and service-development activities in different ways. To distinguish involvement from formally evaluated impact, we describe the topics brought to the groups, the perspectives or recommendations provided by members, and, where documented, subsequent decisions or changes made by the research or clinical teams. No formal evaluation of the impact of these contributions was conducted. During the reporting period, the scope of the Patient Panel broadened beyond the qualitative study through which it was initially established. Subsequent meetings included development of a clinical group intervention, discussion of completed study findings and their implications for the clinical service, and consideration of further research questions arising from these activities. The NDD Advisory Board similarly expanded from discussion of the initial qualitative study to questions brought by researchers working across different areas of neurodevelopmental research. Examples of contributor input and subsequent responses are presented in Table 2.

**Table 2:** PPI activities, contributor input, and subsequent responses during the first year

Structure / session	Activity/topic	Input from PPI contributors	Subsequent response/change and status
Patient Panel – Session 1	Development of the interview guide for the qualitative study of autistic adults’ experiences of accessing psychotherapy	Panel members reviewed the relevance and comprehensibility of interview questions and commented on terminology, question wording and topics. Members could also identify questions/topics that should be added or reformulated.	The final interview guide introduced an explanation of key concepts, elicited each interviewee’s preferred terminology, and instructed interviewers to use that terminology throughout the interview. The final guide added prompts to establish specific previous healthcare encounters and instructed interviewers to anchor follow-up questions in a concrete episode described by the interviewee.
Patient Panel – Session 2	Preliminary findings from the qualitative interview study; development of a clinical group intervention	Panel members discussed the preliminary findings and reviewed the proposed structure and content of a newly designed group intervention for autistic patients. They emphasized the importance of including a more resource-oriented perspective on health and well-being, rather than focusing only on difficulties and psychiatric symptoms.	In response to the Panel’s input, a new Health and Well-being module with a resource-oriented focus was added to the intervention.
Patient Panel – Session 3	Final findings from the qualitative study and implications for clinical service development	Final study findings from the study presented at Session 1 were presented to the Panel. The unit manager also provided an update on the clinical service, including resource allocation.	The discussion contributed to further consideration of how suicide-risk assessment could be adapted for autistic adults. A potential new research project is being explored to develop an autism-sensitive adaptation of structured suicide-risk assessment.
NDD Advisory Board – Session 1	Ethical and methodological issues in the qualitative study conducted within adult autism psychiatry	Board members raised concerns regarding clinician–researcher dual roles, voluntariness and potential power asymmetries, and discussed ways of making the interview procedure more accessible for autistic participants. Recommendations included allowing additional processing time and breaks and providing alternative means of supporting communication during interviews.	The interview procedure was revised to include explicit opportunities for pauses and breaks, additional processing time, paper and pen as a communication support, an explanation of the interview agenda before questioning, and prioritisation of essential questions.
NDD Advisory Board – Session 2	Three researcher-submitted topics: physical activity and problematic school absenteeism; transition from child/adolescent to adult healthcare; early parent–infant interaction/support before autism diagnosis	Board members discussed collaboration between healthcare and education, continuity across service transitions, and family needs for support before formal diagnosis.	Presenting researchers documented feedback relevant to their own projects. They were encouraged to report back to the Board regarding subsequent use of the feedback; no such follow-up had occurred during the reporting period.

Note. NDD Advisory Board = Advisory Board at University of Gothenburg, open to all researchers from Sahlgrenska Academy Faculty with a focus on neurodevelopmental disorder research

The Patient Panel and NDD Advisory Board operated as separate advisory structures, and no formal mechanism was established for transferring recommendations between them. However, some research activities were discussed in both groups. Most notably, the qualitative study in adult autism psychiatry was considered by both structures from different perspectives. The Patient Panel focused primarily on the relevance, comprehensibility, terminology, and content of the interview guide, whereas the NDD Advisory Board focused on ethical and methodological issues, including clinician–researcher roles, voluntariness, power asymmetries, and communication accessibility. In this way, the two structures provided complementary input into different aspects of the same research project.

### Implementation feedback

Following the second NDD Advisory Board meeting, all attendees were invited by email to complete a brief questionnaire intended to support the ongoing development of the Board. The questionnaire was administered as internal implementation feedback rather than as a research instrument. Individual responses or quotations are therefore not reported or analysed as stakeholder research data. The feedback was used as contextual information informing the researchers’ reflections on implementation, particularly regarding preparation, follow-up, and clarity of purpose.

No corresponding structured implementation feedback was collected from Patient Panel members during the reporting period. Panel members expressed positive views about their involvement informally during meetings; however, these comments were neither systematically elicited nor documented for research or evaluation purposes and are therefore not treated as data in the present report. Consequently, we do not draw conclusions about Panel members’ experiences, satisfaction, or perceived value of their involvement.

## Discussion

This contribution describes the first year of implementing two recurring PPI structures within adult autism psychiatry and neurodevelopmental research. Although both structures became operational during the reporting period, they followed different implementation trajectories. The Patient Panel was initially anchored to a specific research programme and subsequently broadened to include intervention development, interpretation of research findings, and clinical service-development questions. In contrast, the NDD Advisory Board was established from the outset as a broader forum in which researchers could bring different neurodevelopmental research projects and questions for discussion.

These different trajectories appeared to reflect, at least partly, the institutional contexts in which the structures were established. Within the clinical setting, establishing the Patient Panel required the development and repeated adjustment of procedures concerning recruitment, handling of contact information, compensation, facilitation, and organizational responsibility. Anchoring the Panel initially to an existing research programme provided a pragmatic route through which these procedures could be established. The university-based Advisory Board could be given a broader remit from the outset and organized more readily through existing academic and organizational networks. Our experience therefore suggests that recurring PPI need not follow a single implementation pathway. In some settings, particularly where involvement is embedded within clinical services, a project-linked starting point may provide an organizational foundation from which broader involvement can subsequently develop.

A second lesson concerned the importance of preparation for meaningful involvement. Contributors need sufficient information to understand the issue under discussion and the type of input being sought. Within the NDD Advisory Board, presenting researchers were asked to formulate specific questions in advance, and project and agenda information was circulated before meetings. This provided a defined basis for discussion and reduced the need to use limited meeting time to introduce unfamiliar projects. Preparation should therefore be regarded as part of the involvement process itself rather than merely as an administrative activity surrounding meetings. However, preparation also raises questions of resources and equity. Written materials provided in advance require contributors’ time: Patient Panel members were compensated for meeting attendance but not for preparation time, while participation in the NDD Advisory Board was uncompensated. Expectations regarding preparation should therefore be proportionate to contributors’ time and the resources available to support their involvement.

The two structures provided different forms of expertise. The Patient Panel primarily contributed experiential knowledge grounded in autism and psychiatric care, whereas the NDD Advisory Board provided a broader forum for methodological and ethical reflection involving researchers and representatives of patient and user organizations. These differences reflected their composition, institutional settings, and mechanisms for determining meeting content rather than two equivalent versions of the same participatory model. The structures operated independently during the first year, and no formal exchange of recommendations or joint meetings took place. Although the same qualitative study was discussed in both structures, the discussions occurred separately and addressed different aspects of the study.

In both structures, involvement remained predominantly advisory. Agendas were largely determined by researchers or clinical staff, and final decisions remained with researchers, project leads, or clinical management. Contributors nevertheless influenced specific decisions. Patient Panel input, for example, resulted in the addition of a resource-oriented Health and Well-being module to the clinical intervention, while recommendations from the NDD Advisory Board were incorporated into communication and accessibility procedures for the qualitative interview study. These examples demonstrate influence on research and service-development activities but should be distinguished from shared decision-making or co-production, neither of which was established during the reporting period. No directly contradictory recommendations between the two structures were documented, and no formal procedure for resolving disagreements between them had been established.

A further lesson concerned the distinction between providing opportunities for input and establishing accountability for how that input is subsequently used. Neither structure used a standardized procedure for recording recommendations, subsequent decisions, and implementation status. Feedback was provided to the Patient Panel at later meetings where relevant, including presentation and discussion of findings from the qualitative study. Researchers presenting to the NDD Advisory Board were encouraged to return and describe how they had used the Board’s input, but such follow-up had not yet occurred during the reporting period. In retrospect, a simple recommendation-and-response procedure would have strengthened transparency and accountability by documenting contributor recommendations, subsequent decisions, reasons where recommendations were not adopted, implementation status, and feedback provided to contributors.

These experiences also require consideration of our own roles in the implementation process. As researchers involved in establishing the structures, we were simultaneously involved in selecting topics, participating in or facilitating discussions, implementing some subsequent changes, and preparing the present account. This proximity enabled some contributor input to be incorporated relatively directly into ongoing research and clinical activities, but it also concentrated authority within the research and clinical teams. Contributors did not formally determine meeting agendas or share final decision-making authority. Greater contributor involvement in agenda setting, governance, evaluation, and reporting therefore represents an important direction for further development.

Our initial ambition to establish recurring rather than project-specific involvement also shaped the Patient Panel. The first year modified our understanding of how such continuity can be achieved. Within the clinical setting, a broad recurring structure could not simply be established by designation; it required workable arrangements for recruitment, data protection, compensation, facilitation, and organizational responsibility. Starting from an existing research programme provided a pragmatic organizational anchor from which the Panel’s remit could subsequently broaden. Sustainability may therefore be better understood as an organizational capacity that develops over time rather than as a characteristic that can be assumed when a PPI structure is established.

The clinical embedding of the Patient Panel created both opportunities and particular power considerations. Its close relationship with the autism psychiatry service allowed lived-experience perspectives and research findings to enter direct dialogue with clinical management and service-development questions. At the same time, Panel members were recruited from a service from which they received psychiatric care, while researchers and clinical staff retained formal decision-making authority. Maintaining a clear distinction between voluntary PPI involvement and the clinical relationship is therefore particularly important in this setting. The involvement of NSPH as an external organization in recruitment contact and meeting moderation provided some organizational separation but did not remove the underlying asymmetry between service users and the healthcare organization.

Accessibility was addressed pragmatically rather than through a formalized procedure. The Patient Panel offered hybrid participation, scheduled breaks, and different modes of providing feedback, while the NDD Advisory Board was conducted online and additional email feedback was encouraged. However, individual communication, sensory, or other accessibility needs were not systematically assessed. Providing a digital option or allowing written feedback should not in itself be equated with accessible involvement. A more proactive approach would include asking contributors about communication and sensory preferences, providing predictable information in advance where possible, and periodically reviewing whether meeting procedures create barriers to participation.

Representation likewise needs to be interpreted in relation to the purpose and recruitment pathways of each structure. The Patient Panel was intentionally clinically anchored, providing experiential perspectives directly relevant to the participating service. This clinical specificity also narrowed representation: the clinic serves autistic adults with co-occurring psychiatric conditions without intellectual disability, and the Panel therefore did not include autistic people with intellectual disability or autistic people outside specialist psychiatric care. The Panel should consequently not be understood as representing autistic adults more broadly. The NDD Advisory Board, in contrast, incorporated perspectives partly through representatives of established patient and user organizations. Such representation can provide valuable collective and advocacy perspectives but should not be assumed to capture the diversity of individual lived experiences.

Terminology itself emerged as an additional area for reflection. The term NDD reflected the institutional and clinical terminology under which the Advisory Board was established and was not formally co-developed with contributors. Internal implementation feedback subsequently raised questions about its terminology and scope. This experience suggests that the language used to name and define participatory structures is not neutral: it may shape how their remit is understood and who perceives themselves as represented or invited to contribute. Terminology should therefore be revisited with contributors as the structures develop.

When considered in relation to the AASPIRE practice-based guidelines [10], implementation was uneven across principles. Recurring opportunities for involvement, collaboration with patient and user organizations, compensation within the Patient Panel, and some flexibility in modes of communication were established during the first year. Other principles were only partially implemented. Accessibility needs were accommodated where possible but were not systematically assessed; compensation practices differed between the structures; and formal procedures for documenting recommendations and reporting subsequent decisions remained limited. The clearest gaps concerned governance and shared decision-making: contributors did not share formal decision-making authority, and involvement in priority setting, data analysis, authorship, and governance remained limited or absent. We therefore regard the AASPIRE guidelines as a reference point for identifying both progress and areas requiring further development rather than as a participatory model achieved by either structure.

### Limitations

Several limitations should be considered when interpreting this account. First, this paper presents a researcher-led reflective account of the establishment and first year of two PPI structures. The authors were directly involved in establishing and operating the structures and, in some cases, in the research and clinical activities discussed within them. Although this proximity provided detailed knowledge of the implementation process, it may also have influenced which experiences were prioritized and how they were interpreted. The manuscript was not co-produced with public contributors, which further limits the extent to which it represents contributors’ own perspectives on the implementation process.

Second, contributors’ experiences of involvement were not systematically evaluated. An internal implementation questionnaire was distributed following the second NDD Advisory Board meeting, but it was intended to support development of the Board rather than as a research instrument and is not analysed as stakeholder research data in this report. No corresponding structured feedback was collected from Patient Panel members. Informal comments made during Panel meetings were likewise not systematically elicited or documented for evaluation purposes. We therefore cannot draw conclusions about contributors’ experiences, satisfaction, acceptability of the involvement process, or perceived value of either structure. In addition, the written format used for the NDD implementation questionnaire may have influenced who chose to respond, as alternative feedback formats were not systematically offered. Future evaluation should offer different modes of providing feedback and, where appropriate, involve public contributors in the evaluation and reporting process.

Third, the reporting period captures only the first year of implementation. The fact that both structures operated during this period does not establish their long-term sustainability or institutionalization. Longer-term organizational responsibility, governance arrangements, resources, and continuity therefore remain to be established.

Fourth, recommendations and subsequent decisions were not prospectively documented through a standardized tracking procedure. Although several concrete changes following PPI activities could be identified, the absence of a systematic recommendation-and-response record limits our ability to attribute individual changes exclusively to particular contributor recommendations or to systematically report recommendations that were not implemented. Future implementation should document recommendations, subsequent decisions, reasons for non-implementation where relevant, implementation status, and feedback provided to contributors.

Fifth, involvement remained predominantly advisory and was concentrated on study design, ethical and methodological reflection, intervention development, interpretation of findings, and service-development questions. Contributors did not share formal decision-making authority, and involvement in data analysis, formal priority setting, governance, and authorship remained limited or absent during the reporting period.

Finally, the two structures captured particular rather than representative perspectives. The Patient Panel was recruited exclusively from one specialist autism psychiatry service and therefore reflected the population served by that clinic: autistic adults with co-occurring psychiatric conditions and without intellectual disability. Consequently, autistic people with intellectual disability and autistic people outside specialist psychiatric care were not represented through this recruitment pathway. The NDD Advisory Board incorporated perspectives partly through representatives of established patient and user organizations; such organizational representation should not be assumed to capture the diversity of individual lived experiences. Demographic characteristics, individual lived-experience characteristics, and accessibility needs were not systematically collected, further limiting our ability to assess the range of perspectives represented or identify other groups whose perspectives may have been absent.

### Practical implications

Several practical lessons emerged during the first year. First, the route to recurring PPI may differ across institutional settings. In the clinical setting, anchoring the Patient Panel initially to an existing research programme provided a practical starting point from which its remit could broaden, whereas the university-based Advisory Board could adopt a broader cross-project remit from the outset. Second, collaboration with established patient and user organizations can provide important organizational capacity. NSPH supported recruitment, meeting organization, and independent moderation of the Patient Panel. However, institutional support alone was insufficient: procedures for handling contact information, compensation, coordination, and organizational responsibilities had to be established and negotiated within the clinical context. Close collaboration with unit and clinic management, and their continued support for embedding participatory research practices within the service, proved important for establishing the Patient Panel in practice. Third, preparation should be considered part of meaningful involvement. Providing contributors with accessible information and clearly formulated questions can support focused discussion, but expectations regarding preparation should be proportionate to contributors’ time and available compensation.

Fourth, recurring involvement requires mechanisms for accountability and follow-up. A simple procedure documenting recommendations, subsequent decisions, implementation status, and feedback to contributors could make the use of PPI input more transparent. Finally, accessibility, roles, and decision-making authority should be clarified from the outset and revisited over time. Contributors and researchers should have a shared understanding of the intended level of involvement and how contributor input can influence subsequent decisions. The first-year experience demonstrates that recurring PPI activities can be established within both clinical and academic settings but does not establish their long-term sustainability or institutionalization. Sustained PPI is likely to depend on organizational support, resources, accessible participation, transparent follow-up, and clearly defined roles and responsibilities.

## Supplementary Information

Below is the link to the electronic supplementary material.

Supplementary Material 1

## Data Availability

No datasets were generated or analysed during the current study.
