# Supplementary material for: Building recurring public and patient involvement in adult autism psychiatry and neurodevelopmental research: lessons from the first year of a patient panel and advisory board
Source: Res Involv Engagem. 2026 Oct 1;12:161. doi: 10.1186/s40900-026-00990-6 (PMC13632358; doi:10.1186/s40900-026-00990-6)
Supplement: Supplementary file 1 [file 40900_2026_990_MOESM1_ESM.docx]

**GRIPP2 Long Form**

| **GRIPP2-LF item** | **Page** |
| --- | --- |
| **1a Aim** | p. 2 |
| **1b Methods** | p. 2 |
| **1c Results** | p. 2 – descriptive PPI contributions and documented subsequent changes; no formal impact evaluation |
| **1d Conclusions** | p. 2 |
| **1e Keywords** | p. 3 |
| **2a Definition** | p. 6 |
| **2b Theoretical underpinnings** | pp. 6–7 |
| **2c Concepts/theory development** | pp. 6–7 – AASPIRE used as a reflective reference framework |
| **3 Aim** | p. 7 |
| **4a Design** | pp. 7–13 |
| **4b People involved** | pp. 8–13, Table 1 |
| **4c Stages of involvement** | pp. 14–18, Table 2 |
| **4d Level/nature of involvement** | pp. 15–20, Tables 1–2 |
| **5a Qualitative evidence of impact** | Not applicable – stakeholder feedback was not subjected to formal qualitative analysis |
| **5b Quantitative evidence of impact** | Not applicable – no quantitative assessment of PPI impact was conducted |
| **5c Robustness of measure** | Not applicable – no formal measure of PPI impact was used |
| **6 Economic assessment** | Not applicable – no formal economic assessment was conducted |
| **7a Outcomes of PPI** | pp. 15–18, Table 2 – documented contributions and subsequent responses/changes; not formally evaluated outcomes |
| **7b Impacts of PPI** | Not formally assessed. Documented subsequent changes are described on pp. 15–18 and Table 2 |
| **7c Context of PPI** | pp. 14–15, 19–24 |
| **7d Process of PPI** | pp. 14–24 |
| **7ei Theory development** | Not applicable – no new PPI theory was developed |
| **7eii Evaluation of theoretical models** | Not applicable – no theoretical model was formally evaluated |
| **7f Measurement** | Not applicable – no PPI impact instrument was developed or tested |
| **7g Economic assessment** | No formal economic assessment; compensation/resource issues described on pp. 13–14 and 19–24 |
| **8a Outcomes** | pp. 19–24 – descriptive reflection on documented contributions and limitations |
| **8b Impacts** | No formal impact evaluation; documented influence and its limitations discussed on pp. 19–24 |
| **8c Definition** | pp. 21–22 |
| **8d Theoretical underpinnings** | p. 22 – reflection against AASPIRE; no theoretical development claimed |
| **8e Context** | pp. 19–24 |
| **8f Process** | pp. 19–24 |
| **8g Measurement/capture of impact** | Not applicable – PPI impact was not formally evaluated or measured |
| **8h Economic assessment** | Not formally assessed; resource and compensation implications discussed on pp. 19 and 24 |
| **8i Reflections/critical perspective** | pp. 19–24 |

PPI=patient and public involvement
